# Discovery of Siderophore and Metallophore Production in the Aerobic Anoxygenic Phototrophs

**DOI:** 10.3390/microorganisms9050959

**Published:** 2021-04-29

**Authors:** Steven B. Kuzyk, Elizabeth Hughes, Vladimir Yurkov

**Affiliations:** Department of Microbiology, University of Manitoba, Winnipeg, MB R3T 2N2, Canada; umkuzyks@myumanitoba.ca (S.B.K.); elizabeth.hughes@umanitoba.ca (E.H.)

**Keywords:** aerobic anoxygenic phototrophs, siderophore, metallophore, CAS assay, *Chromocurvus halotolerans* strain EG19

## Abstract

Aerobic anoxygenic phototrophs have been isolated from a rich variety of environments including marine ecosystems, freshwater and meromictic lakes, hypersaline springs, and biological soil crusts, all in the hopes of understanding their ecological niche. Over 100 isolates were chosen for this study, representing 44 species from 27 genera. Interactions with Fe^3+^ and other metal(loid) cations such as Mg^2+^, V^3+^, Mn^2+^, Co^2+^, Ni^2+^, Cu^2+^, Zn^2+^, Se^4+^ and Te^2+^ were tested using a chromeazurol S assay to detect siderophore or metallophore production, respectively. Representatives from 20 species in 14 genera of α-*Proteobacteria*, or 30% of strains, produced highly diffusible siderophores that could bind one or more metal(loid)s, with activity strength as follows: Fe > Zn > V > Te > Cu > Mn > Mg > Se > Ni > Co. In addition, γ-proteobacterial *Chromocurvus halotolerans*, strain EG19 excreted a brown compound into growth medium, which was purified and confirmed to act as a siderophore. It had an approximate size of ~341 Da and drew similarities to the siderophore rhodotorulic acid, a member of the hydroxamate group, previously found only among yeasts. This study is the first to discover siderophore production to be widespread among the aerobic anoxygenic phototrophs, which may be another key method of metal(loid) chelation and potential detoxification within their environments.

## 1. Introduction

Iron (Fe) is an essential element for life. In microorganisms, it is used as a co-factor for enzymatic processes, such as in electron transfer during respiration and photosynthesis, nucleic acid or chlorophyll synthesis, nitrate reduction, nitrogen fixation and detoxification of oxygen radicals [1,2,3]. The use of Fe in bacteriochlorophyll (BChl) synthesis and the process of photophosphorylation makes it particularly important to phototrophic organisms. Aerobic anoxygenic phototrophs (AAP) are one such physiological group that uses photosynthesis in oxic conditions as an additional energy source to respiration [4]. They make up a significant proportion of many bacterial communities from a host of environments [5], and therefore likely require a substantial Fe uptake. In addition, Fe may be crucial to protect the cells from oxidative stress due to singlet oxygen formation during BChl *a* synthesis. While necessary for metabolism, biologically active Fe is typically quite sparse in nature as its soluble level is very low at soil and water surfaces [2]. In response to this limitation, both bacteria and fungi have developed siderophores to compete for the available Fe [6]. Siderophores, aptly named from the Greek root representing “iron-bearing” [7], are low weight molecules (no more than 1500–2000 Da and generally lower than 1000 Da) with a high-affinity for Fe [2,3,8,9,10]. The molecules are often short polypeptides with modified or D-amino acids [2,9,11]. They can also be made from dicarboxylic acids and diamine or amino alcohols, linked by amide and ester bonds. These building blocks retain some characteristics of amino acids [2]. Siderophores can be classified into two main functional groups. The first is the hydroxamate group, which involves hydroxamic acid and is produced by both fungi and bacteria. Second is the catechol group, compounds of which contain catechol rings and are only produced by bacteria [6,9,10]. Smaller groups such as the hydroxyacids and the α-hydroxy carboxylates are only rarely used by bacteria [9,10]. Usually, siderophores are synthesized and secreted by cells under iron-deplete growth conditions [8]. Once bound to iron, the complex is taken up by the cells in a substrate specific process [2].

While the main purpose of siderophores is Fe acquisition, they may also play some additional roles. In *Pseudomonas aeruginosa*, pyoverdine controls virulence factor production [12]. *Escherichia coli* can be protected from oxidative stress by the catechol type siderophore enterobactin [13]. Pyochelin in *P. aeruginosa* has a toxic effect on eukaryotic cells, possibly aiding in the bacterium’s virulence [13]. Watasemycins, sideromycins, oxachelin and fusigen have antibacterial activity, which may aid in community competition by preventing other populations from growing [13]. Additionally, siderophores have been shown to bind more than one metal [3,14], including some that have higher affinity for Cu or Zn rather than Fe [15,16]. This broad-spectrum activity has required the classification “metallophore” [17,18], which is a term used for secondary metabolites capable of binding a range of metal(loid) cations. When a metallophore would have a specific metal affinity, it would have a sub-categorical name, where siderophore is for Fe-binding, chalkophore for Cu, or zincophore for Zn, all named when discovered.

This concept, of metallophores capable of capturing multiple metal cations has implicated usefulness in toxic heavy metal tolerances. Particularly in extreme environments, where metals can be present at elevated concentrations that inhibit a variety of life [19,20]. Many metal ions can diffuse freely through the cellular membrane, which is inhibited if the metal is bound to a siderophore that is too large to move without active transport. Furthermore, membrane receptors specific to siderophore-iron complexes can differentiate between those containing substitute cations, causing the cell to reject the alternative metal-containing siderophore. The reduction of free metal concentrations in proximity of the bacterium and decreased passive diffusion of unwanted metals into the cell will lower their overall toxic effect [3]. This could be a compelling concept, as AAP possess very high levels of resistance to toxic heavy metal(loid) oxides [21]. While internal enzymatic reduction takes place [22], the external production of siderophores may provide an additional layer of defense. Additionally, as mentioned above, the siderophores’ ability to reduce reactive oxygen species could help AAP as they need protection against oxidative stress due to their aerobic production of BChl *a*.

*Chromocurvus halotolerans* EG19, is a γ-proteobacterial AAP that was isolated during the spring of 2002 from floating microbial mats within the East German Creek System, Manitoba, Canada [23]. As this is a landlocked hypersaline spring system, it likely contains highly endemic communities of microorganisms that have not been mixed with or affected by allochthonous populations [23]. EG19 forms motile, short rod or longer curved rod-shaped, orange-pink bacteria. When grown with complex carbon sources, EG19 produces a brown pigmented hydrophilic compound, which is excreted into the growth medium. While a similar phenomenon had never been reported in other AAP, it was hypothesized that the compound could be a siderophore [24], as ferric bound siderophores can be visually yellow-brown or red-brown [25]. Our study confirms the identity of this extracellular product and describes it as the first siderophore discovered in an AAP. Other AAP from a vast array of environments that do not pigment growth medium were also tested for their production of siderophores, as most of these metal chelating small molecules are colourless, and synthesis is therefore possible.

## 2. Materials and Methods

### 2.1. Bacterial Strains and Growth Conditions

For this study, 101 strains of AAP originating from an assortment of environments, as well as phylogenetically diverse throughout numerous proteobacterial clades were selected from Dr. Vladimir Yurkov’s vast collection. A complete list of chosen strains, original source of isolation, relatedness to type species, and 16S rRNA partial gene sequence accession numbers are listed in Table A1 and Table A2.

Freshwater AAP were cultivated on rich organic (RO) medium as described [26], with one minor modification. Bactopeptone was reduced to 0.5 g/L and casamino acids were supplemented at 0.5 g/L, which provided a larger variety of complex nutrients. Marine AAP requiring salt were propagated on RO medium described above supplemented with 2% NaCl. AAP originally isolated from the East German Creek System, Manitoba, Canada, were grown using medium A (MA) [23]. Those isolated from biological soil crusts of Sandy Lands Forest and Spruce Woods National Park were cultured on Biological Soil Crust Medium A or B (BSCA or BSCB) [27]. Strains from meromictic Mahoney Lake in British Columbia, Canada were grown on N1 medium [28].

In addition to bacterial isolates formerly described elsewhere, some of those chosen for siderophore testing had not been previously published. AAP isolated from the meromictic Blue Lake in British Columbia, Canada, were cultured using a Blue Lake medium (BLM) containing (g/L): MgSO_4_, 0.5; NH_4_Cl, 0.3; KH_2_PO_4_, 0.3; KCl, 0.3; CaCl_2_, 0.05; NaCl, 12.0; NaHCO_3_, 0.5; Na-acetate, 1.0; malic acid, 1.0; yeast extract, 1.0; bactopeptone, 0.5; with vitamins and trace elements solutions, 2.0 mL each; autoclaved at pH 5.9 and then adjusted after autoclaving to pH 7.5 with 0.5 N NaOH.

Furthermore, several AAP had been recovered from the freshwaters of Lake Winnipeg whilst the habitat was under study [29,30]. Specifically, in the spring of 2017, strain AJ72 was collected at Grand Beach from littoral water, AM19, AM27 and AM91 were from littoral sediment of Victoria Beach. During that summer, BA23 and BE100 were isolated at limnetic sites S1 and S5, respectively, while BL67 and BK61 originated within the littoral water and sediment of Grand Beach and Victoria Beach, respectively. Fall samples of Grand Beach sediment contained CK155 and CK182, while Victoria Beach littoral waters revealed isolate CL63. All were purified on the slightly modified freshwater RO medium as described.

### 2.2. Iron Chelating Chromeazurol S Assay

Every chosen strain was grown on 2% agar plates containing their specific growth medium supplemented with the dye chromeazurol S (CAS), which turns blue when bound to Fe and reverts to yellow/orange being released [31]. To make media, 60.5 mg CAS were dissolved in 50 mL ddH_2_O, then mixed with 10 mL of an iron solution containing 1 mM FeCl_3_ and 10 mM HCl. HDTMA (72.9 mg) was dissolved in 40 mL ddH_2_O prior to mixing with the CAS/iron solution, bringing the total volume to 100 mL. Separately, 900 mL of each growth medium (MA, RO, RO 2% NaCl, BSCA, or BLM) were prepared without the addition of iron, but with the correct amount of components for 1 L, as that would be the final volume of medium after combining with CAS/iron solution. Media were then autoclaved at pH 5.9, separate from the CAS mixture. After autoclaving, each medium was adjusted to pH 6.8 and the CAS solution was added. Agar plates would turn blue when solidified. If measurements of ingredients were not exact or added in an incorrect order, CAS would precipitate out of solution and plates with pH higher than 6.8 would appear green instead of blue. CAS plates were heavily inoculated with each strain, and streaked to achieve isolated colonies after 5 days of growth at 28 °C in the dark.

### 2.3. Variant Cation Chromeazurol S Assay

Due to the principle chemistry behind the siderophore assay [31], which used negatively charged CAS dye that would weakly bind the cation Fe^3+^, we decided to replace Fe^3+^ with different metal(loid) cations, including Mg^2+^, V^3+^, Mn^2+^, Co^2+^, Ni^2+^, Cu^2+^, Zn^2+^, Se^4+^ and Te^2+^. Microbial growth with an aura of colour change in medium from blue to yellow hue would signify that microbially produced siderophore could bind the substituted cation, causing the released dye to revert to its yellow colour. These variant cation CAS plates were successfully created as explained in the previous section, cured to diverse shades of blue dependent on the metal(loid), and were screened for metallophore production identically as in the Fe-chelation CAS assay. All cations were purchased from Sigma Aldrich, USA as chloride salts, which were soluble once acidified as described.

### 2.4. Phylogeny

To assess the phylogenetic position, 16S rRNA gene sequences were either acquired from previously published sources, or newly sequenced in this study. A list of accession numbers to partial 16S rRNA gene sequences attained from public repositories was provided in Table A1 and Table A2. From unsequenced strains, DNA was extracted via the phenol chloroform method [32], and sequenced using Sanger technique [33]. Primer set 27F and 1492R, 5′-AGAGTTTGATCCTGGCTCAG-3′ and 5′-GGTTACCTTGTTACGACTT-3′, respectively, was used to achieve a total contiguous 16S rRNA gene sequence length of >1400 bp per strain. New sequences of 16S rRNA genes from previously unidentified AAP strains were deposited to GenBank under accession numbers (MW970346–MW970408) as listed in Table A1 and Table A2. Genetic relation of 16S rRNA gene sequences acquired from each AAP isolate were compared to the archived sequences of type species using the web-based software Basic Local Alignment Search Tool, BLAST [34]. Phylogenetic trees were constructed via MEGA X software [35] with 1000 bootstraps [36], using Maximum Likelihood method to align all AAP 16S rRNA gene sequences to one another based on the General Time Reversible model [37]. Initial tree(s) for the heuristic search were obtained automatically by applying Neighbor-Join and BioNJ algorithms to a matrix of pairwise distances estimated using the Maximum Composite Likelihood (MCL) approach, and then selecting the topology with superior log likelihood value.

### 2.5. Siderophore Isolation and Concentration from C. halotolerans

**Figure 1:**
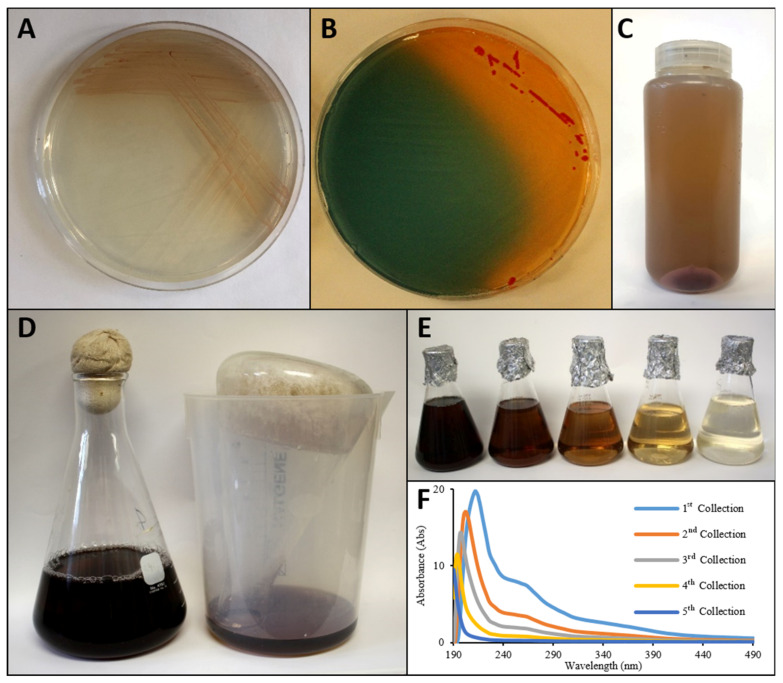
Extraction and concentration of brown pigment excreted by *C. halotolerans*. (**A**) Strain EG19 released a brown pigment into MA medium. (**B**) Fe-CAS plate with siderophore production by EG19. (**C**) Pelleted cells under supernatant. (**D**) Pigment concentrated with freezing-out technique. (**E**) Collected fractions during thawing with concentrated pigment released first. (**F**) Absorbance spectrum of each fraction.

Strain EG19 was grown at 28 °C on MA agarized (2%) plates in the dark for 5 days. Pink-purple colonies developed with brown hue dispersed in agarized medium (Figure 1A), which corresponded to zone of clearing on Fe-Chromeazurol S Assay (Figure 1B). To obtain liquid cultures, EG19 was inoculated at 5% in Fe-free MA and grown for 5 days at 28 °C shaking at 200 rpm in the dark. Fe-free MA was prepared as described in Chromeazurol S Assay, excluding Fe from the trace element solution. In addition, after all components were added, 5 g/L of Chelex resin enclosed in dializing tubing was placed in the medium for 1 h prior to autoclaving. This step allowed the Chelex resin to remove traces of Fe introduced through the complex nutrients such as yeast extract, bactopeptone and casamino acids [38].

After the cultivation of *C. halotolerans*, Fe-free medium became dark brown. The pink-purple cells were pelleted in ~450 mL bottles at 10,000 rpm for 30 min using a Beckman J2HS centrifuge and a JA-10 rotor (Figure 1C). Siderophore containing supernatant was collected and 1.25 L was transferred into 2 L Erlenmeyer flasks (Figure 1D) to freeze overnight at −20 °C. Highly concentrated dark brown-pigmented high-salt slurry was formed predominantly on top of ice, while the lower part of ice was close to colourless. The flask with frozen material was removed from freezer and allowed to defrost at room temperature inserted upside down in a new collection beaker. Thawed supernatant was fractioned into 250 mL batches, with concentrated siderophore thawed and collected first, leaving frozen medium behind (Figure 1D), both observable visually (Figure 1E), and from the absorbance spectrum (Figure 1F).

A batch-type method of siderophore purification was used in this study [39]. Here, the combined freeze-concentrated samples were adjusted to pH 6.0 and XAD 7-HP resin was added (20 g resin per L of supernatant). This slurry was shaken at 200 rpm for 1 h on a rotary shaker and then filtered through a glass Millipore filter funnel that would collect resin, but allow supernatant to easily flow through. Concentrated siderophore required several extractions with resin to remove all pigment from supernatant. Siderophore-bound-resin (now brown in colour) was thoroughly washed with ddH_2_O to remove all residual salts and other soluble contaminants. Vacuum assisted drying using Millipore system was performed, ddH_2_O discarded, and the cleaned siderophore-bound-resin was soaked in methanol for 30 min to release the siderophore. Pigmented methanol was collected, and resin was soaked once more using the same batch of solvent for additional extraction. Methanol extracts were combined and then evaporated to dryness. The resulting dark brown powder was the presumed highly concentrated siderophore, and kept at −20 °C until further testing. Unbound-resin was washed with methanol, then soaked with ddH_2_O prior to reuse.

### 2.6. Purification and Fe-Chelation of Siderophore from C. halotolerans

Concentrated siderophore powder from resin purification was resuspended in ddH_2_O (10 mg in 50 μL), and decimally diluted up to 10^−3^. In addition, a solution containing 5 mg of concentrate in 100 μL ddH_2_O was filtered through an Amicon^®^ Ultra 0.5 mL 3000 Da spin column manufactured by Millipore Ireland, which removed any contaminating proteins over that size. This filtered concentrate was also decimally diluted up to 10^−2^. Once the dilutions were made, 10 μL of each sample, and an aliquot of freeze–thawed concentrate, were individually mixed with 10 μL of loading buffer, prior to filling into the wells of a Mini-Protean tris-tricine gel, 16.5% from Bio-Rad, Hercules, CA, USA. Loading buffer contained 200 mM tris-HCl, pH 6.8, 2% SDS, 40% glycerol and 0.04% Coomassie Brilliant Blue G-250 (CBB) from Bio-Rad, USA. Running buffer was made up of 1 M tris, 1 M tricine and 1% SDS at pH 8.3. Wells were 30 μL, filled with 20 μL of sample (10 μL of sample, 10 μL of loading buffer). Protein ladder was a C6210—color marker ultra-low range (M.W. 1060–26,600) from Sigma-Aldritch, St. Louis, MO, USA. Gel electrophoresis was run at 100 volts for 1 h prior to being stained in a CBB solution (1 g of CBB dissolved in 1 L of [50% MeOH, 10% glacial acetic acid, 40% H_2_O]). The gel was then destained with a mixture containing 5% acetic acid and 20% MeOH overnight.

Fe chelation was tested with the concentrated siderophore powder from the resin concentration step, the brown pigment filter-purified below 3 kDa, as well as with a sample from the remaining proteins that were >3 kDa from the spin filtration procedure. Each fraction was solubilized, where 10 mg of dried pigment was dissolved in 500 µL of 60% MeOH, prior to applying each solution into a blank diffusion disk, manufactured by Oxoid in the UK, allowing it to become dry and concentrated within the disk. These were then placed on a Fe-CAS plate and left to react with chromeazurol S overnight.

## 3. Results

### 3.1. Bacterial Growth and Fe-CAS-Plate Reactions

**Figure 2:**
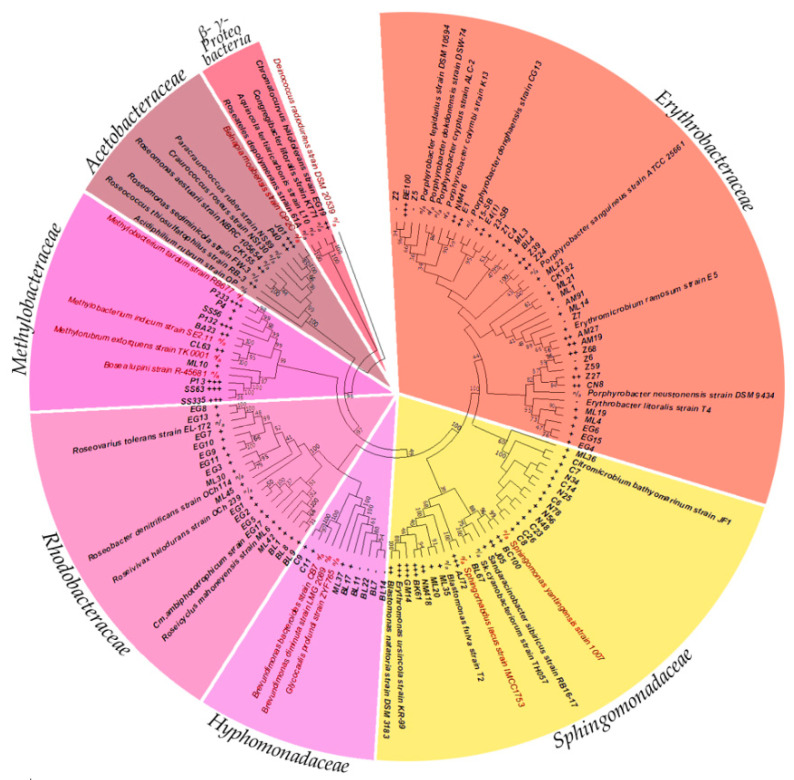
Phylogenetic tree of AAP tested for siderophore production. Isolates hailed from α-proteobacterial families *Erythrobacteraceae*, *Sphingomonadaceae*, *Acetobacteraceae*, *Rhodobacteraceae*, *Hyphomonadaceae* and *Methylobacteraceae*, as well as a representative within the γ- subclass of *Proteobacteria* (titled sections around circumference of circle). Fe-chelation siderophore activities are listed between strain names and phylogenetic position. Strain names in bold are confirmed AAP, red highlighted are not.

**Figure A1:**
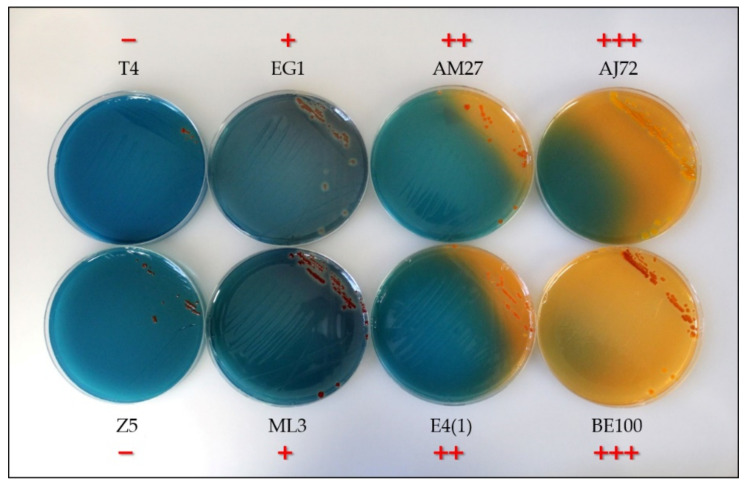
Iron chelating CAS assay after 5 days’ growth. Zones of activity are separated into 4 groups based on range of colour change, as indicated in red by each strain name.

Comparative phenotypic analysis was achieved by allowing each strain to develop for 5 days on their respective agarized media under conditions that promoted the best growth. This elapsed time ensured stationary phase was reached for each representative, which resulted in the formation of sufficient colonies for analysis. Triplicate CAS-supplemented and CAS-free controls were simultaneously plated to identify the viability of inoculum. In all cases, growth occurred on CAS plates, but was marginally reduced in comparison to controls. Both after 3 and 5 days, the average zone of colour change (blue to yellow) was recorded, revealing several phenotypic attributes. As some strains grew slower than others, 5 days’ period was chosen to analyze and compare all simultaneously. We identified siderophore production based on zone of diffusion/colour change around colonies as follows (Figure A1): no zone (−), a zone <1 mm around colonies (+), a moderate diffusion <10 mm (++), and considerable diffusion >10 mm (+++). Fe-chelating CAS reactions after 5 days are listed in Table 1 and Table 2, as well as shown in Figure 2 beside each strain name.

### 3.2. Substitute Cation CAS Assays

**Figure A2:**
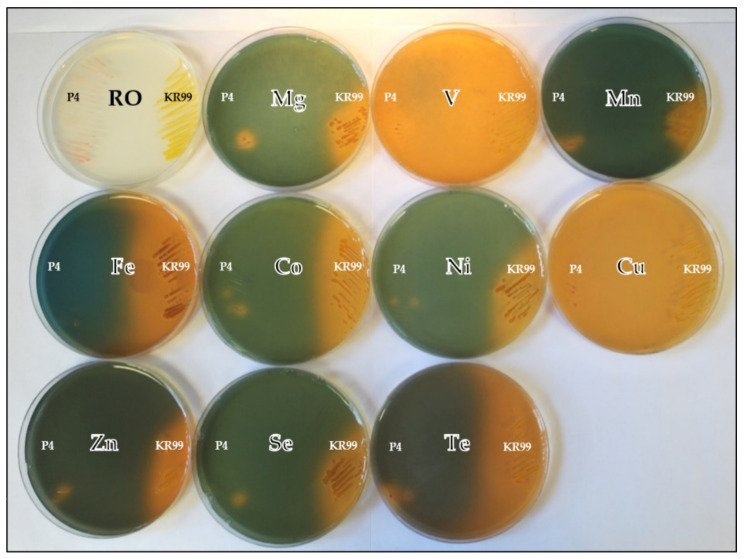
Variant metal(loid) CAS assay after 5 days’ growth for strains P4 and KR99. Growth on RO without CAS was compared to those CAS plates supplemented with Mg, V, Mn, Fe, Co, Ni, Cu, Zn, Se, or Te.

Once Fe-chelating siderophore production was confirmed using the standard Fe-CAS assay, all strains were tested on CAS supplemented agar plates that contained one of 9 other metal(loid) cations: Mg^2+^, V^3+^, Mn^2+^, Co^2+^, Ni^2+^, Cu^2+^, Zn^2+^, Se^4+^ and Te^2+^. Results for varied CAS assays are listed along-side Fe^2+^ data in Table 1 and Table 2 using the same zone distinctions as described. While some strains only produced siderophores that reacted with Fe^2+^, others had secondary metabolites capable of chelating additional metal(loid) cations. Most strains capable of chelating metals other than Fe could also chelate iron, with the exception of two strains P4 and SS56 (Figure A2), which were found to produce metallophore capable of acting predominantly on other tested metal(loid)s, rather than Fe.

### 3.3. Phylogenetic Diversity of Siderophore Producing AAP

Isolates were chosen to represent AAP from a variety of environments as well as embody a host of phylogenetically diverse species. In this way, the 101 representatives listed in Table 1 and Table 2 were cultivated, and 16S rRNA gene sequences acquired either from repositories, or decoded in this work. Phylogenetic relation to sequences of known type strains was determined by BLAST search (Table A1 and Table A2). In addition, these sequences were used to create a phylogenetic tree (Figure 2), that also included some previously described type species not tested for siderophore production, but were included as key placeholders of phylogenetic groups. The evolutionary analysis performed on Mega X using Maximum Likelihood method involved 132 nucleotide sequences and had a total of 1717 positions in the final data set. The chosen AAP diversely spread throughout *Erythrobacteraceae* and *Sphingomonadaceae* relating to known AAP type species. While some aligned to reported AAP in *Acetobacteraceae*, and *Rhodobacteraceae*, many others aligned to organisms previously undescribed as AAP within these clades, as well as to some species within *Hyphomonadaceae* and *Methylobacteraceae*.

### 3.4. C. halotolerans Pigment Purification and Identification

**Figure 3:**
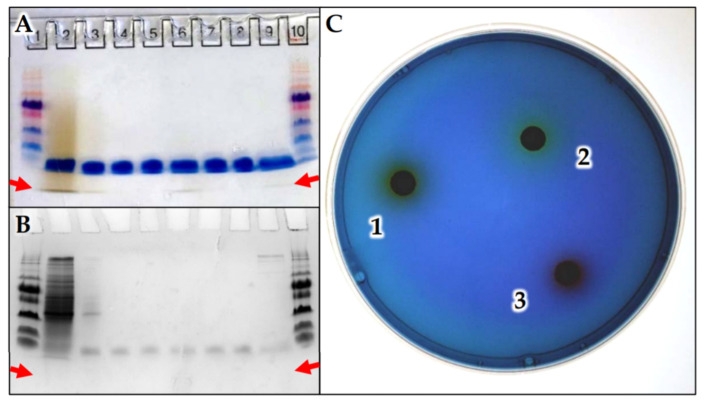
Gel purification of *C. halotolerans* brown pigment, and confirmation of siderophore activity. (**A**) Unstained and (**B**) stained tris-tricine gel electrophoresis performed on: siderophore from resin concentration, wells 2–5; siderophore sample smaller than 3 kDa, wells 6–8; proteins larger than 3 kDa and remaining in solution well 9; standard ladder, wells 1 and 10. (**C**) Siderophore activity observed for: (1) Crude resin extract positive reaction visible as yellowing area; (2) <3 kDa dark brown fraction produced positive yellowing reaction; (3) Proteins >3 kDa negative results observed as darkening of blue due to alkaline pH, without siderophore activity present.

When purified via 16.5% tris-tricine gel electrophoresis, the brown pigment migrated further than the loading buffer’s running dye, CBB, after 1 h (Figure 3A). This gel-shift revealed the pigment under study to be smaller than CBB, which has a known molecular weight of 856.03 g/mol. Since well 1 contained a standard ladder, the measurement of migration distance for the siderophores’ brown band, and of each protein in the ladder allowed for the rough estimation of pigment size to be near ~341 Da (Figure S1). Gel staining and destaining revealed that the siderophore sample collected after resin concentration and diluted in methanol at 200, 20, 2, and 0.2 µg/mL loaded into wells 2 through 5, respectively, had some contaminating small proteins as expected (Figure 3B). In addition, samples that received the subsequent removal of proteins larger than 3 kDa via spin-column were also run on the same gel. Wells 6 through 8 contained brown pigment, which passed through the <3 kDa spin column and then diluted in 60% methanol to 100, 10, and 1 µg/mL, respectively. This step purified the brown pigment of any contaminating proteins (Figure 3A,B). Often, small molecules below 1 kDa are lost from the gel during destaining step [40], which likely caused the small brown pigment to escape similarly to CBB, (Figure 3B). Regardless, testing the crude dried pigment (Disk 1), as well as fractions <3 kDa and >3 kDa (Disks 2 and 3), confirmed the smaller fraction containing brown pigment acted as a siderophore, while the larger proteins did not (Figure 3C). Since the <3 kDa fraction had no contaminants (Figure 3B), but contained the brown pigment prior to destaining (Figure 3A), the small ~341 Da molecule produced by *C. halotolerans* acted as a siderophore.

## 4. Discussion

### 4.1. Siderophore Production Revealed by CAS-Assay

While effectively employed for the identification of numerous siderophore producing bacteria, the CAS assay has a notable limitation; microbial growth may be hindered due to a few factors [31]. Since the metal cation of interest weakly bound to the dye, it was less freely diffusible into the bacteria, resulting in lower availability. In addition, the medium needed to be at pH 6.8 for the indicative colour change to occur successfully. Finally, HDTMA has been known for its slight bacterial toxicity. If an organism had weak Fe transport, required a basic or acidic optimal pH, or was sensitive to HDTMA, it would of had reduced growth. In our experiments, we found that while a range of media compositions could be used, all AAP did have reduced growth on CAS plates, when compared to control. However, since growth did indeed occur, siderophore production could therefore be analyzed. 

Other, more high-throughput, alternative methods were considered, but the chosen agarized CAS-assay was most ideal for determining siderophore and metallophore production. Recently, a bulk screening assay for siderophore detection was proposed [41]. However, both the “traditional” and “modified microplate” qualitative techniques described could not be used for our application, since there was an assumption that siderophores were always constitutively expressed, whereas cultures were grown in complex media without manipulating the concentration of any metal of interest. While this may be the case for some strains, the expression/production of most siderophores or other secondary metabolites usually requires induction from an external factor, which can be either the presence or absence of a stimuli [18]. For biologically significant metals, including Fe, Zn, and Cu, the related cation-specific metallophores are expected to be produced only under limiting conditions. In opposite, metallophores that act on V, Te, Se, or other more toxic metal(loid)s are presumably only synthesized when such toxins are present at higher concentrations. Therefore, growth on agarized plates that contained each metal of interest pre-bound to CAS-reagent as a stimulant for metallophore production was our chosen method. Future works are required to test varying concentrations of metal cations to determine which yields more/less production of specific metallophores. 

Regarding the Fe-chelation, a range of phenotypes was observed, when detecting siderophore activity after the 5-day incubation (Figure A1). In particular, 4 phenotypes were distinguished among all isolates based on size of clearing/colour change zone. Negative results (−) had to have bacterial growth, but without a change in medium opacity. The smallest zone of clearing (+), seen previously after prolonged growth [42], was likely not due to siderophore production [43]. Rather, this small aura could be due to high rates of metal uptake from the surrounding medium. Prolonged bacterial metabolism allowed for increased simple metal diffusion into cells, which reduced its amount in the near-by medium, rendering the dye in that narrow area void of metal, turning yellow. Hence, there is a very small <1 mm zone. In comparison, moderate or highly diffusible siderophore release was quite evident, and was segregated into two phenotypic groups. A zone <10 mm compared to a zone >10 mm, where each represented less or more diffusible secondary metabolite, respectfully, and where lower or higher concentrations were produced and captured additional metal cations. In this test, all 4 zonal varieties for Fe-chelation were discovered (Table 1 and Table 2, and Figure 2).

**Figure 4:**
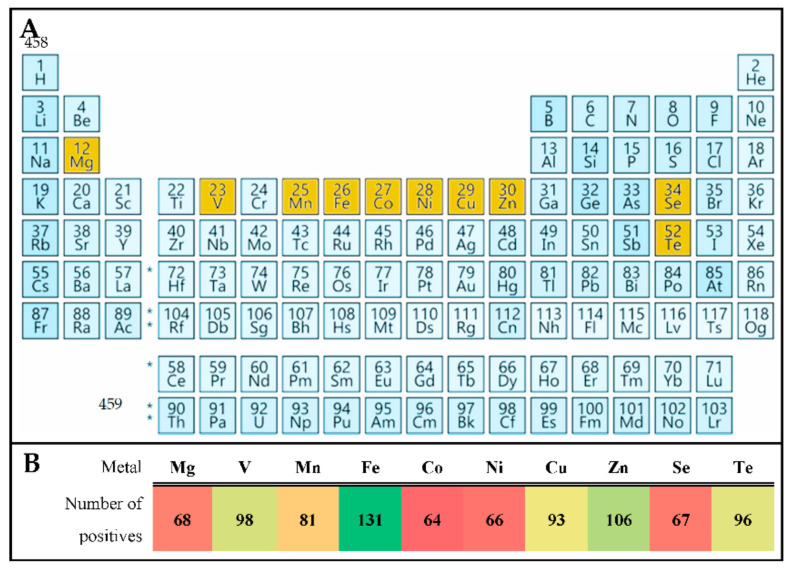
Variant metal(loid)s tested in CAS assay. (**A**) Chosen elements highlighted in yellow. (**B**) Tallied number of positive results from Table 1 and Table 2.

Modifying the CAS assay to monitor chelation of elements other than Fe^2+^ included Mg^2+^, V^3+^, Mn^2+^, Co^2+^, Ni^2+^, Cu^2+^, Zn^2+^, Se^4+^ and Te^2+^ (Figure 4A). The method could be successfully adapted for all selected metal(loid)s, where only Mn^2+^, Co^2+^, Ni^2+^, Cu^2+^, Zn^2+^ had been proposed previously [44]. In addition, the assay could be used with variable nutrient and organic carbon concentrations that did not inhibit the activity. Furthermore, we discovered that a wide range of AAP produced metallophores, which bound a variety of metal(loid)s in addition to Fe (Table 1 and Table 2). Some AAP had siderophores that specifically bound Fe only, including strains E1, E4(1), RB3, NM416, AM27, CK155, BC100, Z24, Z39, and J05. Most AAP with highly diffusible siderophores, and >10 mm zones on Fe-CAS plates, had activity towards a large variety of metal(loid)s. Few strains, including SS56 and P4 preferentially bound metal(loid)s other than Fe. Since all 9 additional cations Mg^2+^, V^3+^, Mn^2+^, Co^2+^, Ni^2+^, Cu^2+^, Zn^2+^, Se^4+^ and Te^2+^ could be bound by metallophores, future work may consider a broader range of cations and the extent of metals that can be exogenously chelated. To determine if specific metal(loid)s were more readily chelated than others, all positive CAS assay results were tallied for each, Figure 4B. Here, multiple +++ represented strong production or significant interaction, and could be compared to weaker reactions such as +, or ++. In this way, Fe was the highest cumulative acquired cation, with the activity ranked as Fe > Zn > V > Te > Cu > Mn > Mg > Se > Ni > Co. It would appear slight variation in cation size, from Fe (55.85) to Co (58.93), had a strong impact on activity, where Fe was most frequently captured, and Co the rarest. Indeed, since strongest reactions existed for Fe and Zn, with less reactivity found for metals in between the sizes of these two within the periodic table, specific mechanisms likely existed to capture either metal, inferring cation specificity for each metallophore. Further analysis will be required to see if the broad range metal(loid) acquisition is due to the production of a single siderophore that reacts with a variety of metals, or the bacteria produced specific metallophores for each. AAP metallophore production could be explained by requirement of trace elements and the need of toxicity prevention. Both Fe and Zn are biologically necessary and commonly limited or unavailable in dissolved forms, therefore acquisition via specific siderophores would be an asset. The 3rd and 4th highest captured metal(loid)s were V and Te, which have known toxic properties, and could have been sequestered as a result of a protection mechanism alone. Bound to a metallophore, these cations would be too large to freely diffuse through outer membranes and be restricted from entering the cell, to prevent any toxic influence. The remaining 6 had reduced activity, likely because they are less toxic and easier available in microbial environment, and therefore less metallophores could be expected.

### 4.2. Environmental Distribution of Siderophore Producers

In relation to the origin of isolation a few patterns were observed. Collections of AAP strains that originated from hot springs, freshwater lakes, and biological soil crusts all had a high proportion of siderophore producers. In opposition, the isolates from marine, meromictic lake, and saline spring habitats produced less, or no siderophores. Rather, they seem to rely on sufficient metal uptake directly from the local microenvironment by diffusion. The main differentiating factor in this case is the requirement of NaCl. It appears that AAP capable of halotolerance or halophilic growth do not produce siderophores of equal activity or quantity as bacteria that do not depend on NaCl for growth. This correlation may be due to each strains’ reaction to osmotic pressure. Cells that are adapted to tolerate higher levels of NaCl will likely have additional cation pumps to survive naturally and resist the high levels of solutes in a saline environment. As seen elsewhere, cation pumps can be non-specific, where many are capable of removing several toxic cations from the cytoplasm of microorganisms [45]. In comparison, freshwater AAP are comparably less osmophilic, and would therefore have less need of copious cation pumps in membranes. Therefore, they are more likely to evolve defensive mechanisms that modify the local environment to suit their needs, including the production of external small molecules that would render cations less diffusible.

### 4.3. Phylogenetic Diversity of Fe-Chelating AAP

Comparing phylogenetic diversity and the production of siderophores on CAS plates by AAP delivered a few notable trends (Figure 2). Broadly, no studied representatives of the order *Rhodobacterales* or *Hyphomonadaceae* had siderophores, as no or <1 mm zone of colour change was present. The *Acetobacteraceae* that were in closest relation to known type species of AAP, strains RB-3T and CK155 had siderophores that only chelated Fe as discussed above. In comparison, strains P40 and J01 were more genetically distant from known type species of AAP, and also produced significant zones of clearing, ≥10 mm for all metal(loid)s tested, signifying their own group. The predominantly strong expression of siderophores by AAP among the *Methylobacteraceae* warrant further study as most type species in this clade have not been previously recognized as phototrophs. Of note, previous research had found that isolates relating to *Methylobacterium mesophilicum* and *M. extorquens* produced siderophores, but the activity had not been linked to aerobic anoxygenic phototrophy [46]. One clade of AAP which related by 98.7–99.1% 16S rRNA sequence similarity to *Bosea lupini*, including strains P13, SS335, and SS63 were all capable of strong production of metallophores, >10 mm zones for multiple metal(loid)s. In comparison, strains P4 and SS56, which have 99.6% relation to *M. phylloshaerae* and 99.6% to *M. branchiatum*, respectively, both had stronger reactions against metal(loid)s that were not Fe (Figure A2). This activity may be explained through the findings of related works, where methanotrophs produced methanobactin, a chalkophore, which is a Cu specific metallophore [15]. The isolates we have tested may indeed possess similar mechanisms as they sequester Cu strongly, as well as Mg, V, Mn, Zn, and Te more favorably than Fe.

*Sphingomonadaceae* could be separated into 3 groups, as those related to *Citromicrobium* did not produce siderophores, *Sphingomonas* relatives produced some siderophores that were predominantly Fe specific, while relatives of *Blastomonas* could produce siderophores that acted on all metal(loid)s tested. A few *Sphingomonas* relatives had been previously found to produce siderophores against Fe [47], but not other metal(loid)s. The *Blastomonas/ Erythromonas* grouping was of particular interest as most representatives revealed strong metallophore production against all 10 cations tested. Our results corresponded well with previous analysis of *E. ursincola*, strain KR99, which had very high resistance to V, Te, and Se oxides, internally reducing them to elemental states [48]. Since KR99 can both acquire Se, Te, and V via metallophore activity (Figure A2), and internally reduce metal(loid) oxides, it appears to require them in reduced form for some reason. Future study of such associations will determine if *E. ursincola* sequesters these cations as a protective measure, or uses them for some metabolic purpose. In comparison, the *Erythrobacteraceae* were not as concisely divided as other families, where those closest to *Erythromicrobium* had siderophores, but most *Porphyrobacter* had very small <1 mm or negligible zones. An exception was strain BE100, which branched distantly from its nearest relative *P. colymbi* (Figure 2), and showed a significant presence of metallophores, ~10 mm zones for all metal(loid)s except Te. Finally, strain EG19 that hailed from γ- rather than α-*Proteobacteria*, had moderate siderophore production for both Fe and Zn, <10 mm. While many siderophores have been discovered as products of bacteria within the γ -proteobacterial clade (Table S1), none have been documented as highly pigmented.

### 4.4. Analysis of the Brown-Coloured Siderophore

Gel purification of brown pigment produced by *C. halotolerans* (Figure 3), revealed that while CBB bound to proteins remained in the gel, both unbound CBB and brown pigment were released during destaining process. Comparing lanes 2–5, the sample prepared by resin concentration clearly contained brown pigment, but also accumulated proteins smaller than 26 kDa. The use of the 3 kDa cut off spin column did indeed remove these contaminants, as shown in lanes 6–8. Since the small brown pigment passed through the spin column, was purified on gel electrophoresis, and maintained activity on CAS plates, these tests confirmed that it acted as a siderophore. Furthermore, these procedures established the brown compound to clearly be under 800 Da, and approximated to be around ~341 Da when correlated to the ladder during TRIS-tricine gel electrophoresis (Figure S1). Both the estimated small size and the brown appearance of the siderophore synthesized by *C. halotolerans* were useful for its tentative identification. The most comparable small molecule described in literature was rhodotorulic acid (Table S1), a 344.4 Da siderophore that was pigmented red when bound to Fe [49]. However, this acid has only been naturally found in yeasts including *Rhodotorula pilimanae*, with no known bacterial producers [50]. With that in mind, hydroxamic acids are produced by both bacteria and fungi [51,52], and therefore similar secondary metabolites can be expected in other species. In addition, the colour disparity, red compared to orange-brown, may indicate an altered structure among siderophores produced by *R. pilimanae* and *C. halotolerans*, respectively.

Since *C. halotolerans* hails from the γ-*Proteobacteria*, comparisons must be drawn between its siderophore and those produced by other species in the γ-subclass. The most similar in size was acinetobactin, a 346.4 Da molecule from *Acinetobacter baumannii* expressed using the operon containing *basABCDEFGHIJ*, *bauABCDEF* and *barAB* genes [53]. *C. halotolerans* genome, published within the One Thousand Microbial Genomes Phase 4 (KMG IV) project by the DOE Joint Genome Institute, submitted online in 2019 with accession number PRJNA520330 [54], contained neither similar genes nor was the operon present, while using very low homology search. Due to the divergence between *C. halotolerans*’ pigmented siderophore and *A. baumannii*’s lack of colour, and the absence of similar genes, we assume that acinetobactin was not the siderophore of *C. halotolerans*. Further structural analysis will be necessary to confirm the structural identity of this novel compound.

## 5. Conclusions

We have discovered that many AAP produce siderophores or metallophores as diffusible secondary metabolites. Production could be related to acquisition of metal(loid)s including magnesium, vanadium, manganese, iron, cobalt, nickel, copper, zinc, selenium and tellurium, or to provide resistance to toxic metal(loid)s in environments with elevated concentrations. A correlation existed between site of isolation and production of siderophores, whereas tested freshwater AAP produced siderophores, and AAP that required NaCl predominantly did not. Furthermore, there could be connection between phylogeny of isolates and their ability to form siderophores, but as with many phenotypes to genotype comparisons, it did not appear as a strictly followed rule. With such considerations, siderophore production cannot be recommended as a taxonomic marker for AAP identification, as variable production types occurred. However, a potential application exists to use this phenotypic feature during taxonomic differentiation between species. Future work will hopefully identify the siderophores, and potential metallophores, produced by each AAP, and determine the total list of metal cations that can be targeted.

## Figures and Tables

**Table 1:** Freshwater and saline AAP analyzed for metal(loid)-chelation via CAS assays with varied cations.

Environment	Strain	Medium	Mg	V	Mn	Fe	Co	Ni	Cu	Zn	Se	Te
Hot spring, Kamchatka Isl.	KR99^T^	RO	++	+++	++	+++	++	++	++	+++	++	+++
Warm temperature spring, Baikal Lake, Russia	E1	RO	−	−	+	++	+	+	−	−	−	−
	E4(1)	RO	−	−	−	++	−	−	−	−	−	+
	E5^T^	RO	−	−	−	+	−	−	−	−	−	+
	RB3^T^	RO	−	−	++	++	−	++	+	+	+	+
	RB16-17^T^	RO	−	−	−	+	−	−	+	−	−	−
	T4^T^	RO-NaCl	+	+	+	−	+	+	−	−	+	+
Deep Ocean, Juan De Fuca Ridge, Pacific Ocean	JF1^T^	RO-NaCl	+	+	+	+	+	+	+	+	+	+
	C6	RO-NaCl	+	+	+	+	+	+	+	+	+	+
	C7	RO-NaCl	+	+	+	+	−	+	+	+	+	+
	C8	RO-NaCl	+	+	+	+	−	+	+	+	+	+
	C14	RO-NaCl	+	+	+	+	−	+	+	+	+	+
	C23	RO-NaCl	−	+	+	+	+	+	+	+	+	+
	C26	RO-NaCl	+	+	+	+	+	+	+	+	+	+
	N25	RO-NaCl	+	+	+	+	+	+	+	+	+	+
	N34	RO-NaCl	+	+	+	+	+	+	+	+	+	+
	N48	RO-NaCl	+	+	+	+	+	+	+	+	+	+
	N56	RO-NaCl	+	+	+	+	+	+	+	+	+	+
	N78	RO-NaCl	+	+	+	+	+	+	+	+	+	+
Rag Beach sediment, BC, Canada	15-SB	RO-NaCl	+	−	+	+	−	−	+	+	+	+
	23-SB	RO-NaCl	+	−	+	+	+	+	−	+	−	−
Central Gold Mine, MB, Canada	C4	RO	−	+	+	+	+	+	+	+	−	+
	C9	RO	−	+	−	+	+	−	+	+	−	+
	C11	RO	−	+	−	+	−	−	+	+	−	−
	NM4.16	RO	−	+	+	++	+	+	+	+	−	+
	NM4.18	RO	++	+	+	++	++	++	++	+	++	++
Lake Winnipeg, MB, Canada	AJ 72	RO	−	+	+	+++	+	+	+	++	−	+
	AM 19	RO	−	+	−	+++	−	−	−	++	−	−
	AM 27	RO	−	−	−	++	−	−	−	+	−	−
	AM 91	RO	−	−	−	-	−	−	−	−	−	−
	BA 23	RO	+	++	++	++	++	+	++	++	++	++
	BC 100	RO	−	−	−	++	+	−	−	−	−	−
	BE 100	RO	++	++	++	+++	++	++	++	++	++	+
	BK 61	RO	+	+	+	+++	+	−	+	++	+	−
	BL 67	RO	−	−	−	−	+	−	−	−	−	−
	CK 155	RO	−	−	−	++	−	−	−	−	−	−
	CK 182	RO	−	−	−	−	−	−	−	−	−	−
	CL 63	RO	+	++	++	++	++	++	++	++	++	++
	CN8	RO	+	++	+	++	+	+	−	++	++	+
	GM14	RO	++	++	++	+++	++	+	−	++	++	+
Zebra mussels, Lake Winnipeg, MB, Canada	Z1	RO	−	−	−	+	−	−	−	−	−	−
	Z2	RO	−	−	−	−	−	−	−	−	−	−
	Z5	RO	−	−	−	−	−	−	−	−	−	−
	Z6	RO	+	−	+	−	+	+	−	−	+	−
	Z7	RO	+	−	−	−	−	−	−	−	−	−
	Z24	RO	−	−	−	++	−	−	−	+	-	−
	Z27	RO	+	+	+	++	−	−	−	++	+	++
	Z39	RO	−	−	+	++	+	−	−	+	−	−
	Z59	RO	+	−	+	+	−	+	−	−	+	−
	Z68	RO	−	++	−	++	−	+	−	++	−	+

**Table 2:** Meromictic lake, saline spring and biological soil crust AAP analyzed for metal(oid)-chelation via CAS assays with varied cations.

Environment	Strain	Medium	Mg	V	Mn	Fe	Co	Ni	Cu	Zn	Se	Te
Mahoney Lake, BC, Canada	ML1	N1	−	+	−	+	−	−	+	+	−	+
	ML3	N1	−	+	−	+	−	−	+	+	−	+
	ML4^T^	N1	−	+	−	+	−	−	+	+	−	+
	ML6^T^	N1	+	+	+	+	−	−	+	+	−	+
	ML10	N1	−	+	−	+	−	−	+	+	−	+
	ML14	N1	−	+	−	+	−	−	+	+	−	+
	ML19	N1	+	+	+	+	+	−	+	+	+	+
	ML20	N1	−	+	−	+	−	−	+	+	−	+
	ML21	N1	−	+	−	+	−	−	+	+	−	+
	ML22	N1	−	+	+	+	−	−	+	+	−	+
	ML30	N1	+	+	−	+	−	−	+	+	+	+
	ML35	N1	−	+	−	+	−	−	+	+	−	+
	ML36	N1	+	+	+	+	+	−	+	+	+	+
	ML37	N1	−	+	+	+	−	−	+	+	−	+
	ML42	N1	+	+	+	+	+	+	+	+	+	+
	ML45	N1	+	+	+	+	−	+	+	+	+	+
Blue Lake, BC, Canada	BL1	BLM	+	+	+	+	+	+	+	+	+	+
	BL4	BLM	−	+	−	+	−	−	+	+	−	+
	BL5	BLM	+	+	+	+	+	+	+	+	+	+
	BL7	BLM	+	+	+	−	+	+	+	−	+	+
	BL8	BLM	+	+	+	+	+	+	+	+	+	+
	BL9	BLM	+	+	+	+	+	+	+	+	+	+
	BL11	BLM	+	+	+	−	+	+	+	+	+	+
	BL14	BLM	+	+	+	−	+	+	+	+	+	+
	BL17	BLM	+	+	+	+	+	+	+	+	+	+
	BL22	BLM	+	−	+	−	+	+	+	−	+	+
East German Creek System, MB, Canada	EG1	MA	−	+	−	+	−	−	+	+	−	+
	EG2	MA	+	+	+	+	−	−	+	+	−	+
	EG3	MA	−	-	−	+	−	−	−	−	−	−
	EG4	MA	−	+	−	+	−	−	+	+	−	+
	EG5	MA	−	+	+	+	−	−	+	+	−	+
	EG6	MA	−	+	−	+	−	−	+	+	−	+
	EG7	MA	−	+	+	+	−	−	+	+	−	+
	EG8	MA	−	+	−	+	+	−	+	+	+	+
	EG9	MA	−	+	−	+	−	−	+	+	−	+
	EG10	MA	−	+	−	+	−	−	+	+	+	+
	EG11	MA	−	+	−	+	−	−	+	+	−	+
	EG13	MA	−	+	−	+	−	−	+	+	−	+
	EG15	MA	−	+	−	+	+	−	+	+	−	+
	EG17^T^	MA	+	+	+	+	+	+	+	+	−	+
	EG19^T^	MA	+	+	+	++	−	+	+	++	+	+
Sandy Lands Forest soil crust, MB, Canada	SS56	BSCA	++	++	++	+	++	++	++	++	++	++
	SS63	BSCA	++	+++	++	+++	++	+++	+++	+++	+++	+++
	SS335	BSCA	+++	+++	+++	+++	++	++	+++	++	++	+++
Spruce Woods National Park soil crust, MB, Canada	J01	BSCA	+++	++	+++	+++	+	++	++	++	+	++
	J05	BSCA	+	+	+	++	−	−	+	+	−	+
	P4	BSCB	++	++	++	+	+	+	++	++	++	++
	P13	BSCB	+++	+++	+++	+++	++	+++	+++	+++	++	++
	P40	BSCB	++	++	++	++	+	++	++	++	+	+
	P132	BSCB	++	++	++	+++	++	++	++	++	++	++
	P233	BSCB	−	++	++	+++	++	++	++	++	++	++

## Data Availability

Data is contained within the article.

## Supplementary Materials

The following are available online at https://www.mdpi.com/article/10.3390/microorganisms9050959/s1, Figure S1: Determination of siderophore size, Table S1: Siderophores examples listed from largest to smallest.

## Appendix A

**Table A1:** Freshwater and saline AAP chosen for siderophore testing.

Environment	Strain	Medium	Most Related Type Species	Accession #
Hot spring, Kamchatka island	KR99^T^	RO	*Erythromonas ursincola*	NR_119243.1
Warm temperature spring, Bikal Lake, Russia	E1	RO	*99.6% Porphyrobacter colymbi*	MW970346
	E4(1)	RO	*99.7% Porphyrobacter donghaensis*	MW970347
	E5^T^	RO	*Erythromicrobium ramosum*	NR_041891.1
	RB3^T^	RO	*Roseococcus thiosulfatophilus*	NR_026114.1
	RB16-17^T^	RO	*Sandaracinobacter sibiricus*	NR_026382.1
	T4^T^	RO-NaCl	*Erythrobacter litoralis*	NR_119016.1
Deep Ocean, Juan DeFuco Ridge, Pacific Ocean	JF1^T^	RO-NaCl	*Citromicrobium bathyomarinum*	Y16267.1
	C6	RO-NaCl	*98.1% Citromicrobium bathyomarinum*	MW970348
	C7	RO-NaCl	*99.8%* *Citromicrobium bathyomarinum*	MW970349
	C8	RO-NaCl	*99.9%* *Citromicrobium bathyomarinum*	MW970350
	C14	RO-NaCl	*99.6% Citromicrobium bathyomarinum*	MW970351
	C23	RO-NaCl	*99.7% Citromicrobium bathyomarinum*	MW970352
	C26	RO-NaCl	*99.8% Citromicrobium bathyomarinum*	MW970353
	N25	RO-NaCl	*99.7% Citromicrobium bathyomarinum*	MW970354
	N34	RO-NaCl	*99.7% Citromicrobium bathyomarinum*	MW970355
	N48	RO-NaCl	*99.8% Citromicrobium bathyomarinum*	MW970356
	N56	RO-NaCl	*99.9% Citromicrobium bathyomarinum*	MW970357
	N78	RO-NaCl	*99.6% Citromicrobium bathyomarinum*	MW970358
Rag Beach sediment, BC, Canada	23-SB	RO-NaCl	*99.7% Porphyrobacter donghaensis*	MW970359
	15-SB	RO-NaCl	*99.8% Porphyrobacter donghaensis*	MW970360
Central Gold Mine, MB, Canada	C4	RO	*99.4% Porphyrobacter colymbi*	KX148515
	C9	RO	*99.1% Brevundimonas variabilis*	KX148516
	C11	RO	*98.6% Brevundimonas bacteroides*	KX148517
	NM4.16	RO	*99.7% Porphyrobacter colymbi*	KX148518
	NM4.18	RO	*99.3% Blastomonas fulva*	KX148519
Lake Winnipeg, MB, Canada	AJ 72	RO	*99.7% Sphingorhabdus lacus*	MW970361
	AM 19	RO	*99.7% Erythromicrobium ramosum*	MW970362
	AM 27	RO	*99.6% Erythromicrobium ramosum*	MW970363
	CK 182	RO	*93.4% Porphyrobacter donghaensis*	MW970364
	BL 67	RO	*98.9% Sandarakinorhabdus cyanobacteriorum*	MW970365
	BK 61	RO	*99.1% Blastomonas fulva*	MW970366
	BE 100	RO	*99.4% Porphyrobacter colymbi*	MW970367
	AM 91	RO	*98.5% Porphyrobacter sanguineus*	MW970368
	CK 155	RO	*98.1% Roseomonas sediminicola*	MW970369
	CL 63	RO	*100% Methylorubrum extorquens*	MW970370
	BA 23	RO	*96.2% Methylobacterium indicum*	MW970371
	BC100	RO	*96.6% Sphingomonas yantingensis*	MW970372
	CN8	RO	*99.2% Porphyrobacter neustonensis*	MW970373
	GM14	RO	*99.2% Erythromonas ursincola*	MW970374
Zebra mussels, Lake Winnipeg, MB, Canada	Z1	RO	*99.1% Porphyrobacter colymbi*	MN987006
	Z2	RO	*99.4% Porphyrobacter tepidarius*	MN987007
	Z5	RO	*99.6% Porphyrobacter tepidarius*	MN987008
	Z6	RO	*99.3% Porphyrobacter neustonensis*	MN987009
	Z7	RO	*99.6% Erythromicrobium ramosum*	MN987010
	Z24	RO	*98.7% Porphyrobacter sanguineus*	MN987011
	Z27	RO	*99.4% Porphyrobacter neustonensis*	MN987012
	Z39	RO	*98.9% Porphyrobacter sanguineus*	MN987013
	Z59	RO	*99.5% Porphyrobacter neustonensis*	MN987014
	Z68	RO	*99.4% Porphyrobacter neustonensis*	MN987015

**Table A2:** Meromictic lake and biological soil crust AAP chosen for siderophore testing.

Environment	Strain	Medium	Most Related Type Species	Accession #
Mahoney Lake, BC, Canada	ML1	N1	*98.4% Erythromicrobium ramosum*	MW970375
	ML3	N1	*99.4% Porphyrobacter sanguineus*	MW970376
	ML4^T^	N1	*Porphyrobacter meromictius*	NR_115007.1
	ML6^T^	N1	*Roseicyclus mahoneyensis*	NR_042080.1
	ML10	N1	*97.9% Salinarimonas ramus*	MW970377
	ML14	N1	*98.9% Erythromicrobium ramosum*	MW970378
	ML19	N1	*96.9% Porphyrobacter meromiticus*	MW970379
	ML20	N1	*99.3% Blastomonas fulva*	MW970380
	ML21	N1	*98.5% Erythromicrobium ramosum*	MW970381
	ML22	N1	*99.8% Porphyrobacter sanguineus*	MW970382
	ML30	N1	*98.8% Seohaeicola saemankumensis*	MW970383
	ML35	N1	*99.4% Blastomonas fulva*	MW970384
	ML36	N1	*97.1% Porphyrobacter sanguineus*	MW970385
	ML37	N1	*95.4% Glycocaulis profundi*	MW970386
	ML42	N1	*98.0% Roseicyclus marinus*	MW970387
	ML45	N1	*94.7% Ruegeria intermedia*	MW970388
Blue Lake, BC, Canada	BL1	BLM	*98.1% Roseicyclus marinus*	MW970389
	BL4	BLM	*99.3% Porphyrobacter sanguineus*	MW970390
	BL5	BLM	*99.2% Seohaeicola saemankumensis*	MW970391
	BL7	BLM	*95.6% Glycocaulis profundi*	MW970392
	BL8	BLM	*98.1% Roseicyclus marinus*	MW970393
	BL9	BLM	*98.0% Roseicyclus marinus*	MW970394
	BL11	BLM	*95.8% Glycocaulis profundi*	MW970395
	BL14	BLM	*95.6% Glycocaulis profundi*	MW970396
	BL17	BLM	*95.1% Glycocaulis profundi*	MW970397
	BL22	BLM	*95.0% Glycocaulis profundi*	MW970398
East German Creek System, MB, Canada	EG1	MA	*95.2% Roseovarius pacificus*	AM691094
	EG2	MA	*94.4% Roseovarius bejariae*	AM691093
	EG3	MA	*97.3% Yoonia vestfoldensis*	AM691092
	EG4	MA	*98.8% Erythrobacter longus*	AM691105
	EG5	MA	*95.7% Roseovarius pacificus*	AM691095
	EG6	MA	*98.4% Porphyrobacter meromictius*	AM691106
	EG7	MA	*97.3% Roseovarius nitratireducens*	AM691097
	EG8	MA	*99.0% Roseovarius tolerans*	AM691101
	EG9	MA	*97.3% Roseovarius nitratireducens*	AM691098
	EG10	MA	*98.2% Roseovarius tibetensis*	AM691100
	EG11	MA	*97.7% Roseovarius nitratireducens*	AM691099
	EG13	MA	*99.0% Roseovarius tolerans*	AM691102
	EG15	MA	*98.9% Erythrobacter aquimaris*	AM691107
	EG17^T^	MA	*Charonomicrobium ambiphototrophicum*	AM691091
	EG19^T^	MA	*Chromocurvus halotolerans*	AM691088
Sandy Lands Forest Soil Crust, MB, Canada	SS56	BSCA	*99.6% Methylobacterium brachiatum*	MW970399
	SS63	BSCA	*99.1% Bosea lupini*	MW970400
	SS335	BSCA	*98.3% Bosea lupini*	MW970401
Spruce Woods National Park Soil Crust, MB, Canada	J01	BSCA	*98.4% Belnapia moabensis*	MW970402
	J05	BSCA	*96.6% Sphingomonas pruni*	MW970403
	P4	BSCB	*99.6% Methylobacterium phyllosphaerae*	MW970404
	P13	BSCB	*98.7% Bosea lupini*	MW970405
	P40	BSCB	*99.9% Belnapia soli*	MW970406
	P132	BSCB	*99.6% Methylobacterium brachiatum*	MW970407
	P233	BSCB	*99.8% Methylobacterium tardum*	MW970408

## References

[1] Granger J., Price N.M. (1999). The importance of siderophores in iron nutrition of heterotrophic marine bacteria. Limnol. Oceanogr..

[2] Challis G.L. (2005). A widely distributed bacterial pathway for siderophore biosynthesis independent of nonribosomal peptide synthetases. ChemBioChem.

[3] Schalk I.J., Hannauer M., Braud A. (2011). New roles for bacterial siderophores in metal transport and tolerance. Environ. Microbiol..

[4] Yurkov V.V., van Gemerden H. (1993). Impact of light/dark regimen on growth rate, biomass formation and bacteriochlorophyll synthesis in Erythromicrobium hydrolyticum. Arch. Microbiol..

[5] Yurkov V., Hughes E., Hallenbeck P.C. (2017). Aerobic Anoxygenic Phototrophs: Four Decades of Mystery. Modern Topics in the Phototrophic Prokaryotes: Environmental and Applied Aspects.

[6] Faraldo-Gómez J.D., Sansom M.S.P. (2003). Acquisition of siderophores in gram-negative bacteria. Nat. Rev. Mol. Cell Biol..

[7] Lankford C.E. (1973). Bacterial assimilation of iron. Crit. Rev. Microbiol..

[8] Reid R.T., Livet D.H., Faulkner D.J., Butler A. (1993). A siderophore from a marine bacterium with an exceptional ferric ion affinity constant. Nature.

[9] Wandersman C., Delepelaire P. (2004). Bacterial iron sources: From siderophores to hemophores. Annu. Rev. Microbiol..

[10] Grobelak A., Hiller J. (2017). Bacterial siderophores promote plant growth: Screening of catechol and hydroxamate siderophores. Int. J. Phytoremed..

[11] Crosa J.H., Walsh C.T. (2002). Genetics and Assembly Line Enzymology of Siderophore Biosynthesis in Bacteria. Microbiol. Mol. Biol. Rev..

[12] Lamont I.L., Beare P.A., Ochsner U., Vasil A.I., Vasil M.L. (2002). Siderophore-mediated signaling regulates virulence factor production in Pseudomonas aeruginosa. Proc. Natl. Acad. Sci. USA.

[13] Adler C., Corbalán N.S., Seyedsayamdost M.R., Pomares M.F., de Cristóbal R.E., Clardy J., Kolter R., Vincent P.A. (2012). Catecholate Siderophores Protect Bacteria from Pyochelin Toxicity. PLoS ONE.

[14] Ghssein G., Brutesco C., Ouerdane L., Fojcik C., Izaute A., Wang S., Hajjar C., Lobinski R., Lemaire D., Richaud P. (2016). Biosynthesis of a broad-spectrum nicotianamine-like metallophore in Staphylococcus aureus. Science.

[15] Kim H.J., Graham D.W., DiSpirito A.A., Alterman M.A., Galeva N., Larive C.K., Asunskis D., Sherwood P.M.A. (2004). Methanobactin, a copper-acquisition compound from methane-oxidizing bacteria. Science.

[16] Morey J.R., Kehl-Fie T.E. (2020). Bioinformatic Mapping of Opine-Like Zincophore Biosynthesis in Bacteria. mSystems.

[17] Welch R.M., Shuman L. (1995). Micronutrient Nutrition of Plants. CRC Crit. Rev. Plant Sci..

[18] Pedler J.F., Parker D.R., Crowley D.E. (2000). Zinc deficiency-induced phytosiderophore release by the Triticaceae is not consistently expressed in solution culture. Planta.

[19] Nies D.H. (2000). Heavy metal-resistant bacteria as extremophiles: Molecular physiology and biotechnological use of *Ralstonia* sp. CH34. Extremophiles.

[20] Rothschild L.J., Mancinelli R.L. (2001). Life in extreme environments. Nature.

[21] Csotonyi J.T., Maltman C., Yurkov V. (2014). Influence of tellurite on synthesis of bacteriochlorophyll and carotenoids in aerobic anoxygenic phototrophic bacteria. Res. Trends Photochem. Photobiol..

[22] Maltman C., Yurkov V. (2015). The Effect of Tellurite on Highly Resistant Freshwater Aerobic Anoxygenic Phototrophs and Their Strategies for Reduction. Microorganisms.

[23] Csotonyi J.T., Swiderski J., Stackebrandt E., Yurkov V.V. (2008). Novel halophilic aerobic anoxygenic phototrophs from a Canadian hypersaline spring system. Extremophiles.

[24] Csotonyi J.T., Stackebrandt E., Swiderski J., Schumann P., Yurkov V. (2011). *Chromocurvus halotolerans* gen. nov., sp. nov., a gammaproteobacterial obligately aerobic anoxygenic phototroph, isolated from a Canadian hypersaline spring. Arch. Microbiol..

[25] Drechsel H., Jung G. (1998). Peptide siderophores. J. Pept. Sci..

[26] Yurkov V., Dworkin M., Falkow S., Rosenberg E., Schleifer K.-H., Stackebrandt E. (2006). Aerobic Phototrophic Proteobacteria. The Prokaryotes.

[27] Csotonyi J.T., Swiderski J., Stackebrandt E., Yurkov V. (2010). A new environment for aerobic anoxygenic phototrophic bacteria: Biological soil crusts. Environ. Microbiol. Rep..

[28] Yurkova N., Rathgeber C., Swiderski J., Stackebrandt E., Beatty J.T., Hall K.J., Yurkov V. (2002). Diversity, distribution and physiology of the aerobic phototrophic bacteria in the mixolimnion of a meromictic lake. FEMS Microbiol. Ecol..

[29] Kuzyk S.B., Pritchard A.O., Plouffe J., Sorensen J.L., Yurkov V. (2020). Psychrotrophic violacein-producing bacteria isolated from Lake Winnipeg, Canada. J. Great Lakes Res..

[30] Kuzyk S.B., Wiens K., Ma X., Yurkov V. (2020). Association of aerobic anoxygenic phototrophs and zebra mussels, Dreissena polymorpha, within the littoral zone of Lake Winnipeg. J. Great Lakes Res..

[31] Schwyn B., Neilands J.B. (1987). Universal chemical assay for the detection and determination of siderophores. Anal. Biochem..

[32] Rainey F.A., Ward-Rainey N., Kroppenstedt R.M., Stackebrandt E. (1996). The genus Nocardiopsis represents a phylogenetically coherent taxon and a distinct actinomycete lineage: Proposal of *Nocardiopsaceae* fam. nov. Int. J. Syst. Bacteriol..

[33] Sanger F., Coulson A.R. (1975). A rapid method for determining sequences in DNA by primed synthesis with DNA polymerase. J. Mol. Biol..

[34] Madden T., McEntyre J., Ostell J. (2002). The BLAST Sequence Analysis Tool. The NCBI Handbook [Internet].

[35] Kumar S., Stecher G., Li M., Knyaz C., Tamura K. (2018). MEGA X: Molecular evolutionary genetics analysis across computing platforms. Mol. Biol. Evol..

[36] Felsenstein J. (1985). Confidence Limits on Phylogenies: An Approach Using the Bootstrap. Evolution.

[37] Nei M., Kumar S. (2000). Molecular Evolution and Phylogenetics.

[38] Barker R., Boden N., Cayley G., Charlton S.C., Henson R., Holmes M.C., Kelly I.D., Knowles P.F. (1979). Properties of cupric ions in benzylamine oxidase from pig plasma as studied by magnetic-resonance and kinetic methods. Biochem. J..

[39] Yamamoto S., Okujo N., Sakakibara Y. (1994). Isolation and structure elucidation of acinetobactin., a novel siderophore from *Acinetobacter baumannii*. Arch. Microbiol..

[40] Schägger H., von Jagow G. (1987). Tricine-sodium dodecyl sulfate-polyacrylamide gel electrophoresis for the separation of proteins in the range from 1 to 100 kDa. Anal. Biochem..

[41] Arora N.K., Verma M. (2017). Modified microplate method for rapid and efficient estimation of siderophore produced by bacteria. 3 Biotech.

[42] Ames-Gottfred N.P., Christie B.R., Jordan D.C. (1989). Use of the Chrome Azurol S Agar Plate Technique to Differentiate Strains and Field Isolates of Rhizobium leguminosarum biovar trifolii. Appl. Environ. Microbiol..

[43] Amaro C., Aznar R., Alcaide E., Lemos M.L. (1990). Iron-binding compounds and related outer membrane proteins in Vibrio cholerae non-O1 strains from aquatic environments. Appl. Environ. Microbiol..

[44] Patel P.R., Shaikh S.S., Sayyed R.Z. (2018). Modified chrome azurol S method for detection and estimation of siderophores having affinity for metal ions other than iron. Environ. Sustain..

[45] Rensing C., Ghosh M., Rosen B.P. (1999). Families of Soft-Metal-Ion-Transporting ATPases. J. Bacteriol..

[46] Idris R., Trifonova R., Puschenreiter M., Wenzel W.W., Sessitsch A. (2004). Bacterial communities associated with flowering plants of the Ni hyperaccumulator Thlaspi goesingense. Appl. Environ. Microbiol..

[47] Sun L.N., Zhang Y.F., He L.Y., Chen Z.J., Wang Q.Y., Qian M., Sheng X.F. (2010). Genetic diversity and characterization of heavy metal-resistant-endophytic bacteria from two copper-tolerant plant species on copper mine wasteland. Bioresour. Technol..

[48] Maltman C., Donald L., Yurkov V. (2017). Tellurite and Tellurate Reduction by the Aerobic Anoxygenic Phototroph Erythromonas ursincola, Strain KR99 Is Carried out by a Novel Membrane Associated Enzyme. Microorganisms.

[49] Atkin C.L., Neilands J.B. (1968). Rhodotorulic Acid, a Diketopiperazine Dihydroxamic Acid with Growth-Factor Activity. I. Isolation and Characterization. Biochemistry.

[50] Andersen D., Renshaw J.C., Wiebe M.G. (2003). Rhodotorulic acid production by *Rhodotorula mucilaginosa*. Mycol. Res..

[51] Carson K.C., Meyer J.M., Dilworth M.J. (2000). Hydroxamate siderophores of root nodule bacteria. Soil Biol. Biochem..

[52] Holinsworth B., Martin J.D. (2009). Siderophore production by marine-derived fungi. BioMetals.

[53] Mihara K., Tanabe T., Yamakawa Y., Funahashi T., Nakao H., Narimatsu S., Yamamoto S. (2004). Identification and transcriptional organization of a gene cluster involved in biosynthesis and transport of acinetobactin, a siderophore produced by *Acinetobacter baumannii* ATCC 19606T. Microbiology.

[54] Goeker M. (2016). The One Thousand Microbial Genomes Phase 4 Project (KMG-4) Sequencing the Most Valuable Type-Strain Genomes for Metagenomic Binning, Comparative Biology and Taxonomic Classification.

