# Supplementary material for: Discovery of Siderophore and Metallophore Production in the Aerobic Anoxygenic Phototrophs
Source: Microorganisms. 2021 Apr 29;9(5):959. doi: 10.3390/microorganisms9050959 (PMC8146977; doi:10.3390/microorganisms9050959)
Supplement: Supplementary file 1 [file microorganisms-09-00959-s001.zip › microorganisms-1173760-supplementary.pdf]

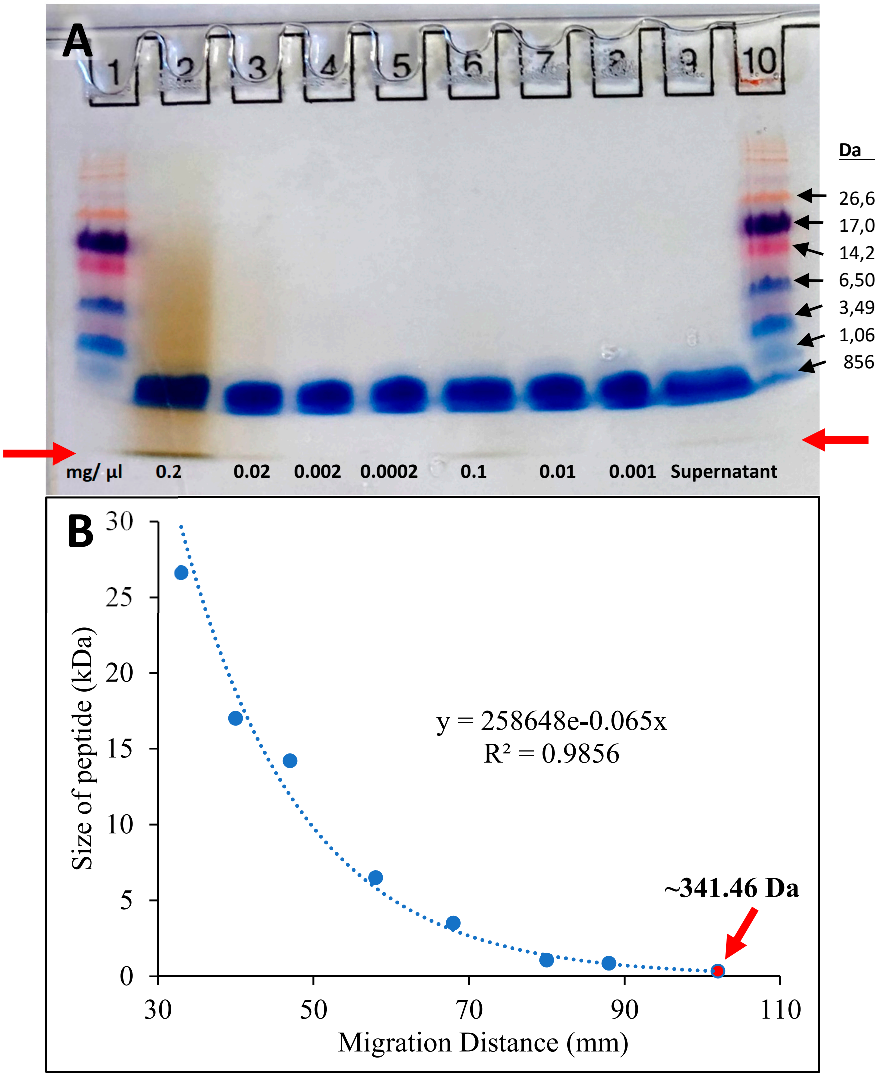

Da	Colour	Protein
26,600	Orange	Triosephosphate Isomerase, rabbit muscle
17,000	Violet	Myoglobin, horse heart
14,200	Red	a-Lactalbumin, bovine milk
6,500	Blue	Aprotinin, bovine lung
3,496	Blue	Insulin Chain B, Oxidized, bovine
1,060	Blue	Bradykinin
856	Blue	Coomassie Brilliant Blue Loading Dye

Figure S1. Approximation of siderophore size. (A) Sample and standard ladder run on tris-tricine gel had migration distances measured, noting size of each known protein. (B) Standard curve of migration distance compared to known sizes used to calculate brown pigment weight of **~341.46 Da**.

Table S1. Siderophores examples listed from largest to smallest.

Siderophore	Size (Da)	Species that produces	Phylum
**Pyoverdine**	1365.4	*Pseudomonadaceae*	*γ-Proteobacteria*
**Alterobactin B**	945.9	*Pseudoalteromonas luteoviolacea*	*γ-Proteobacteria*
**Alterobactin A**	927.9	*Pseudoalteromonas luteoviolacea*	*γ-Proteobacteria*
**Ferrichrome**	740.5	*Aspergillus, Ustilago, and Penicillium*	(Fungi)
**Enterobactin**	669.6	*E. coli and Salmonella typhimurium*	*γ-Proteobacteria*
**Acinetobactin**	346.4	*Acinetobacter baumannii*	*γ-Proteobacteria*
**Rhodotorulic acid**	344.4	*Rhodotorula pilimanae*	(Yeast)
